# Malformations of the craniocervical junction (chiari type I and syringomyelia: classification, diagnosis and treatment)

**DOI:** 10.1186/1471-2474-10-S1-S1

**Published:** 2009-12-17

**Authors:** Alfredo Avellaneda Fernández, Alberto Isla Guerrero, Maravillas Izquierdo Martínez, María Eugenia Amado Vázquez, Javier Barrón Fernández, Ester Chesa i Octavio, Javier De la Cruz Labrado, Mercedes Escribano Silva, Marta Fernández de Gamboa Fernández de Araoz, Rocío García-Ramos, Miguel García Ribes, Carmen Gómez, Joaquín Insausti Valdivia, Ramón Navarro Valbuena, José R Ramón

**Affiliations:** 1 Carlos III Health Institute. Sinesio Delgado n. 6 (pavilion 12), 28029, Madrid, Spain; 2 Spanish Society of Primary Care. c/ Narváez, 15 1º Izda 28009, Madrid, Spain; 3 Spanish Society of Neurosurgery, Spain; 4 Public Health and Health Management Chair, European University of Madrid Villaviciosa de Odón, Spain; 5 Spanish Society of Physiotherapy, Madrid, Spain; 6 Osteopathy, Cantabrian Health Service, Spain; 7 Association of Syringomyelia Sufferers, Barcelona, Spain; 8 Spanish Society of Psychology, Pozuelo de Alarcón, Madrid, Spain; 9 National Association of Friends of Arnold Chiari, Burgos, Spain; 10 Spanish Society of Neurology, Barcelona, Spain; 11 Spanish Society of Family and Community Medicine, Barcelona, Spain; 12 Spanish Society of Medical-Physical Rehabilitation of Madrid, Spain; 13 Spanish Society of Pain, Madrid, Spain; 14 Spanish Society of Pediatric Neurosurgery, Spain

## Abstract

Chiari disease (or malformation) is in general a congenital condition characterized by an anatomic defect of the base of the skull, in which the cerebellum and brain stem herniate through the foramen magnum into the cervical spinal canal. The onset of Chiari syndrome symptoms usually occurs in the second or third decade (age 25 to 45 years). Symptoms may vary between periods of exacerbation and remission. The diagnosis of Chiari type I malformation in patients with or without symptoms is established with neuroimaging techniques. The most effective therapy for patients with Chiari type I malformation/syringomyelia is surgical decompression of the foramen magnum, however there are non-surgical therapy to relieve neurophatic pain: either pharmacological and non-pharmacological. Pharmacological therapy use drugs that act on different components of pain. Non-pharmacological therapies are primarly based on spinal or peripheral electrical stimulation.

It is important to determine the needs of the patients in terms of health-care, social, educational, occupational, and relationship issues, in addition to those derived from information aspects, particularly at onset of symptoms.

Currently, there is no consensus among the specialists regarding the etiology of the disease or how to approach, monitor, follow-up, and treat the condition.

It is necessary that the physicians involved in the care of people with this condition comprehensively approach the management and follow-up of the patients, and that they organize interdisciplinary teams including all the professionals that can help to increase the quality of life of patients.

## Background

For several decades, the eponyms Arnold and Chiari have been used as synonyms to define conditions with ectopia of the cerebellar tonsils below the level of the posterior edge of the foramen magnum. The first case was described by Cleland in 1883. The most detailed original description, however, was made by Chiari in 1891. In the mid-1970s, the term Chiari was again used to name the syndrome.

Chiari syndrome is a developmental malformation of the occipital mesodermal somites that can be associated to syringomyelia and hydrocephalus. The most extreme form consists of the herniation of structures of the lower cerebellum, the cerebellar tonsils, and brain stem through the foramen magnum, in such a way that parts of the brain enter the spinal canal, thickening and compressing it. Symptoms typically appear during adolescence or adulthood, and are not usually accompanied by hydrocephalus. In general, patients complain of recurrent headache, cervical pain, and progressive spasticity of the lower limbs.

Among the many malformations of the craniocervical junction, Chiari type I syndrome and syringomyelia are noteworthy because of their prevalence and the seriousness of their symptom.

The word syringomyelia means reed- or flute-like spinal cord. The disease affects both genders, although with slight predominance in women, and all races. Although most authors consider the average at presentation approximately 35 years [1-5], symptoms onset can occur at any age, from 1 year [6,7] to older than 60 and are highly uncommon in people older than 65 years [8]. In most cases syringomyelia is due to craniocervical malformations, mainly Chiari malformation and basilar impression, so all the problems due to those conditions added to those of syringomyelia, can severely affect the patient's quality of life if adequate and timely measures are not taken.

There are two types of therapies to treat these malformations: surgical therapy, that should be considered for symptomatic patients and which main goal is the decompression of the foramen magnum; and non-surgical therapy, used to relieve the symptoms caused by neuropathic pain.

Although malformations of the craniocervical junction are considered rare diseases (RDs) due to their low incidence, the increasingly frequent use of neuroimaging techniques in clinical protocols has led to such a large increase in the diagnosis of tonsil herniations that there is discussion in the medical literature about whether the prevalence figures known for these diseases should be revised. On the other hand, it is important to develop a consensus regarding the management and the therapeutic approach of these malformations, particularly, considering that in many cases they remain asymptomatic for years.

A classification of the Chiari malformation and related issues about the disease, such as diagnosis and treatment is discussed below, as well as their social and health concernings.

## Classification

The classification of Chiari malformation recognizes five subtypes:

*Chiari type 0 malformation*: characterized by an alteration in Cerebro Spinal Fluid (CSF) hydrodynamics at the level of the foramen magnum. Patients with this subtype have syringomyelia either without tonsil herniation or with only mild tonsil herniation-associated findings.

*Chiari type I malformation*: caudal herniation of the cerebellar tonsils exceeding 5 mm below the foramen magnum. This malformation is typically associated with hydrosyringomyelia. It is not usually accompanied by descent of the brain stem or IV ventricle, nor associated with the presence of hydrocephalus.

*Chiari type II malformation*: caudal herniation of the cerebellar vermis, brain stem, and IV ventricle through the foramen magnum. It is associated with myelomeningocele, hydrocephalus, and, less frequently, hydrosyringomyelia. Other types of intracranial defects (hypoplastic tentorium cerebelli, cranial lacunae, anomalies of the Sylvius aqueduct) may exist.

*Chiari type III malformation*: consists of occipital encephalocoele, with some of the intracranial defects associated with Chiari II malformation.

*Chiari type IV malformation*: cerebellar aplasia or hypoplasia, associated with aplasia of the tentorium cerebelli.

**Figure 1:**
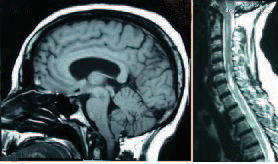
Patient with Chiari I malformation (left) and cervical syringomyelia (right)

Chiari type I malformation is undoubtedly the most frequent subtype. It can coexist with other defects, which are classified according the involved area:

Spinal cord: The most frequent defect associated to Chiari I is syringomyelia. It is agreed that 40-75% of Chiari type I malformations have associated syringomyelia. In contrast, almost 90% of syringomyelias are associated with Chiari malformation. Syringomyelia is a chronic spinal cord defect in which a tubular cavity, or central cavitation, is present in several spinal cord segments  (Figure 1).

The cervical region is the most frequently involved, but the dilation may extend cranially to the brain stem or caudally to the thoracic or lumbar segments. Four types of syringomyelia exist:

*Type I*: syringomyelia with obstruction of the foramen magnum and dilation of the central spinal canal: A) Associated to Chiari type I malformation. B) Associated with other obstructive lesions of the foramen magnum

*Type II*: syringomyelia without obstruction of the foramen magnum, or idiopathic.

*Type III*: syringomyelia with other diseases of the spinal cord. A) Spinal cord tumours (usually intraspinal) B) Traumatic myelopathy C) Spinal arachnoiditis and pachymeningitis D) Myelomalacia due to compression of the spinal cord (tumour, spondylosis)

*Type IV*: Pure hydromyelia, usually associated to hydrocephalus.

Bone malformations of the craniocervical junction occur in about 50% of  the patients with Chiari type I malformation, although the frequency ranges from 45 to 60%, depending on the series.

Posterior fossa volume anomalies are highly significant due to their pathogenic implications. It has been observed that the posterior fossa is narrower and smaller in patients with Chiari malformation than in the general population.

Skull defects sometimes are underdiagnosed if appropriate diagnostic tools are not used. Empty sella turcica, platybasia (flattening of the skull base), basilar impression (elevation of the floor of the posterior fossa with inward displacement of the dens within the occipital foramen), third occipital condyle, and vestiges of the proatlas usually cause anterior compression of the bulbospinal junction, contributing, together with the posterior compression that Chiari malformation causes, to the reduction of the space for the neuroaxis at the level of the bulbospinal junction.

Spine defects: Klippel-Feil anomaly, or fusion of the atlas to the occipital. Dens retroflexion and scoliosis may also be present. Scoliosis has been found in 50-70% of Chiari type II malformations, whereas the association seems to be less frequent in type I malformations, depending on the series. Scoliosis is almost always associated to syringomyelia and has a left curvature, unlike idiopathic scoliosis, which usually is a dextroscoliosis. In cases with syringomyelia, weakness of the spinal axial musculature is due to a progressive disorder of the motor neuron, which results in denervation of the paravertebral muscles.

Ventricles and cisterns: Hydrocephalus only occurs in 3-10% of patients with Chiari type I malformation. In contrast, practically always occur in Chiari type II malformation.

Meninges: Thinning of the meninges occurs at the level of the foramen magnum. Several bands of dura mater constricting the foramen magnum and posterior arch of the atlas are often present. In surgical series, arachnoiditis caused by repeated rubbing of the abnormally herniated cerebellar tonsils against the leptomeninge and dura mater in the foramen magnum has been reported. Arachnoiditis has been confirmed in postoperative histopathology studies and it is believed to increase as the herniation evolves in time.

Brain: In Chiari I malformations, no associated cerebral anomalies exist. The only defect sometimes observed is thinning of the medulla oblongata and loss of folia in the herniated cerebellar tonsils. These findings do not have clinical implications.

There is no universally accepted theory explaining the Chiari malformation and its associated anomalies. Even acquired forms of cerebellar tonsil herniation are recognised. Chiari I and II malformation tend to be more frequent in women and, in some subtypes of Chiari malformation, a genetic factor is beginning to be discussed. Two findings support the existence of a genetic factor: the familial association observed and the coexistence with genetic anomalies (Klippel-Feil or achondroplasia). Families with several members affected have been reported and Milhorat et al [9] found that 12% of patients in their series have a close family member with Chiari type I malformation or syringomyelia. Despite all these cases, no definitive conclusion can be drawn regarding the suspected existence of a genetic factor and the familiar association for this condition.

In the Chiari I-syringomyelia complex, various pathogenic hypotheses are currently postulated, although the most accepted refer to a mechanical factor (excessively small posterior fossa) and an embryonic developmental anomaly [10-15].

## Clinical manifestations

The onset of Chiari syndrome symptoms usually occurs in the second or third decade (age 25 to 45 years), although it is commonly earlier in patients with syringomyelia. Symptoms generally have an insidious onset and a progressive course. There is high clinical variability among patients, ranging from asymptomatic patients, patients with non-specific clinical manifestations, to patients with severe neurologic deficits.

### Symptoms

Symptoms may vary between periods of exacerbation and remission. Suboccipital headache is the most frequent symptom in these patients. The headache is located in the occipital and is of oppressive nature, increasing with Valsalva manoeuvres (such as coughing, sneezing, or bowel movement). The headache can also have non-specific features or a tensional profile.

Neck pain is frequent and characterized by the absence of radicular distribution. It is associated to continuous, burning, deep-seated discomfort in the shoulders, nape, chest, and upper limbs. Neck pain usually increases with Valsalva manoeuvres.

Vertigo can occur, particularly positional vertigo, or triggered by head movements. Other otological symptoms present in these patients are tinnitus and aural fullness. Occasionally, mild neurosensorial hearing loss, with peripheral vestibulopathy is found in otological evaluations

Other frequent symptoms are ocular, often with unremarkable neuro-ophthalmologic examination. The most frequent ocular symptoms are: retro-orbital headache, diplopia, photopsia, blurred vision, and photophobia.

In very severe cases in which compression of the spinal cord or medulla oblongata occurs, symptoms of involvement of the motor or sensory pathways, or lower cranial nerves exist.

Breathing-related sleep disorders were frequently reported in patients with craniocervical junction malformations. Chiari type I malformation should be considered in the differential diagnosis of central apnoeas in infants, especially when they are associated with other neurological sign or symptom. Some authors considered the presence of sleep disorders as an earlier marker of progressive brain stem dysfunction. [16-18]

### Physical signs

Phenotypically, up to 25% of patients may have a short, or bull-like neck. In cases associated to syringomyelia, levoscoliosis may be present. In these patients, the involvement of various nervous structures result in mixed physical signs:

*First motor neuron*: generalised hypereflexia, spasticity, and Babinski reflex, mostly in the lower limbs.

*Second motor neuron*: atrophy, weakness, fasciculations, and areflexia, mostly in the upper limbs.

*Sensory system*: central cord syndrome typical of syringomyelia.

*Cerebellum*: nystagmus, ataxia, and dysmetria.

*Lower cranial nerves:* affected in 15-25% of cases. The following findings may exist: vocal cord paralysis, soft palate weakness, lingual atrophy, cricopharyngeal achalasia, facial hypoaesthesia, and absent gag reflex (the most frequent physical sign indicating involvement of the lower cranial nerves).

## Diagnosis

The delay from the onset of the disease to the diagnosis of any craneocervical junction malformation, before 1985 was significantly longer than after 1985 when magnetic resonance imaging became widely available in clinical practice. That period of time usually was shorther in the pediatric group. [19]

The diagnosis of Chiari type I malformation in patients with or without symptoms is established with neuroimaging techniques; the preferred technique is magnetic resonance imaging (MRI) [20]. MRI can also be used to study the volume of the posterior fossa and CSF flow dynamics. The diagnosis of syringomyelia should be made by MRI of the complete spine (cervical, dorsal, and lumbar). Plain radiography and Computed Axial Tomography (CAT) are used to study bone anomalies. Cranial CAT is also useful for monitoring hydrocephalus.

## Therapy

### Surgical therapy

Asymptomatic patients who are diagnosed of Chiari type I malformation without syringomyelia should not be considered as candidates for surgery. In asymptomatic Chiari type I malformation with syringomyelia, the opinion of neurosurgeons varies. In symptomatic patients, surgical treatment should be considered.

Approximately 10% of patients with Chiari type I malformation have hydrocephalus. Various techniques are used to treat this malformation, but they all involve decompression at the foramen magnum.

As in all surgical procedures, decompression of the foramen magnum in Chiari malformation is not free from complications. Most of them involve CSF disorders, which are usually present in about 10% of patients. These include CSF fistula, meningitis, hydrocephalus, or the progression of syringomyelia. Postoperative relief of preoperative pathologies was experienced in 83% of patients. Of the most common presenting symptoms, headache/neck pain and scoliosis, 12 and 17%, respectively, were not alleviated postoperatively. However, the mortality rate, which is usually due to respiratory arrest in the immediate postoperative period or a serious sequela, should be less than 2%. [21,22]

Most patients experience an improved quality of life after surgery. The symptoms that improve most are mainly headache and neck pain, followed by symptoms attributable to direct compression of the cerebellum or brain stem (e.g., dysphagia, ataxia, nystagmus, and diplopia). In contrast, the symptoms attributable to syringomyelia (pain, scoliosis, and loss of sensitivity) improve less.

If syringomyelia persists, inadequate decompression of the craniocervical junction should be considered. Syringomyelia can reappear in up to 10-20% of patients, due to either inadequate decompression or excessive scar tissue formation, impairing CSF flow.

**Figure 2:**
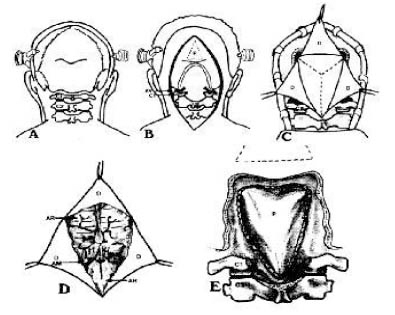
Syringomyelia as a result of fracture of the thoracic spine.

In post-traumatic syringomyelia (Figure 2), some authors [23,24] prefer to restore the canal, which avoids CSF blockade, and empty the cyst or leave a drainage tube in the subarachnoid space. Other authors support conservative treatment [25]. In cases of syringomyelic cysts associated to tumours, cyst reduction is generally achieved by excising the tumour [26,27].

### Non-surgical therapy

#### Analgesia

Neuropathic pain is caused by the lesion of junctional structures between the cerebral base and the cerebellum, and the cervical spinal cord. When a nervous system lesion occurs, different symptoms appear, some are due to loss of function when damage is severe and there is total disruption of nerve conduction; other symptoms are due to irritation (Table 1)

**Table 1:** Symptoms due to loss of funtion, when damage is severe and there is total disruption of nerve conduction. Symptoms due to irritation, when the lesion is less severe or nerve conduction is not totally disrupted.

Symptoms due to loss funtion	Symptoms due to irritation
Motor symptoms	

Paresis Paralysis	Myoclonia Fasciculations Spasticity

Sensory symptoms	

Hypoaesthesia Hypoalgesia Anosmia Amaurosis Deafness Vasodilations Hypo/anhydrosis Loss of pilorection	Paraesthesia Dysaesthesia Allodynia Hyperalgesia Pain Photopsia Tinnitus Vasoconstriction Hyperhyrosis Pilorection

Autonomic symptoms	

Physical findings typical of neuropathic pain can be found, particularly:

Allodynia: a painful response to a non-painful stimulus, such as, brushing the skin with a cotton wad or sponge.

Hyperalgesia: an excessively painful response to a mildly painful stimulus, such as a slight prick.

Pain therapy in Chiari/syringomyelia is problematic, as in any disease with low incidence, in which there is little scientific evidence. Given the high variability in intensity, severity, and location of symptoms, each patient must receive individualized treatment. Globally, there are two types of therapy: pharmacological and non-pharmacological.

##### Pharmacological analgesia

Neuropathic pain should be managed with a multifactorial approach using drugs that act on different components of pain, including disturbed neuronal activity (anticonvulsants [28-32] and local anaesthetics [33]), potentiation of descending inhibitory pathways (antidepressants [34]), or centres involved in the development and conduction of nociceptive responses (analgesics [35-37]). Some of these drugs, their mode of action and side effects are described in Table 2.

**Table 2:** Drugs used to manage neurophatic pain and their mode of action.

Type	Mode of action	Drug	Side effect
Anticonvulsants	Inhibits opening neuronal voltage –dependent channels (calcium channels, sodium channel ) andGABA receptor.	Carbamazepine Gabapentin Pregabalin Topiramate	Hepatotoxicity, drowsiness, fatigue, ataxia, vertigo, gastrointestinal discomfort headache, blurred vision.

Antidepressants	Inhibits the re-uptake of the neurotransmitters norepinephrine and serotonin by neurons.	Amitriptyline Duloxetine Venlafaxine	Mouth dryness, intense sedation, fatigue, diminished libido, weight loss, nausea, insomnia, headache.

Local anaesthetics	Act mainly by inhibiting sodium influx through sodium-specific ion channels in the neuronal cell membrane.	Lidocaine Mexiletine	Dizzine, arrhythmia.

Analgesics	Act through specific receptors, particularly μ receptors distributed throughout the central and peripheral nervous system blocking them.	Tramadol Dextropropoxyphene Buprenorphine Morphine Oxycodone Fentanyl Methadone	Nausea, vomiting, sweating, dizzines, mouth dryness, sedation, vertigo.

Neuropathic pain has traditionally been considered resistant to analgesics, specifically opiates, but it is now admitted that there is some response to these molecules, but less satisfactory than in nociceptive pain. Although the use of most of these drugs is backed by scientific evidence obtained in the treatment of other forms of neuropathic pain, evidence of their effect against the pain produced by Chiari syndrome or syringomyelia is limited due to the low incidence of these conditions and the lack of studies assessing non-surgical treatment for these patients. Some studies suggest that the use of drug combinations, such as weak opiates with anticonvulsants, can be more effective in neuropathic pain and require lower doses.

Spinal infusion of medications has been used for years, but there is little evidence of its efficacy in neuropathic pain and none in Chiari syndrome. Spinal infusion systems comprise an implantable pump system for controlled drug administration, and a catheter through which the medication is directly infused into the cerebrospinal fluid bathing the spinal cord. Implantation of both elements allows prolonged therapy without complications. Drugs used most often with this system include morphine, bupivacaine, clonidine [38], and baclofen [39].

##### Non-pharmacological pain management

While little evidence is available to support pharmacological therapy for pain, there is no evidence at all on the effect of non-pharmacological therapies. Therapies that could benefit these patients are reserved for use when all other therapeutic options fail. Non-pharmacological therapies are primarly based on spinal or peripheral electrical stimulation.

When the symptoms of neuropathic pain are localized in a specific territory, such as one or both upper or lower limbs, electrical stimulation [40] is a potential option. Although spinal stimulation has demonstrated its efficacy in certain types of neuropathic pain, this is not the case for central pain, and there is no evidence available in patients with Chiari syndrome. However, since this therapy is free of major side effects, and any side effect that could occur is reversible, it is an option to be considered before using more questionable therapeutic approaches. Peripheral stimulation has been used recently to treat certain conditions. For example, headache refractory to pharmacologic therapy has been treated with electric stimulation of the occipital nerves. There is little evidence available on the efficacy of this therapy because the technique has been very recently developed, but it could be a therapeutic option for headache in patients with Chiari syndrome if no other treatment has been effective.

#### Rehabilitation

Rehabilitation is a medical specialty that receives patients referred from both primary care and specialized care. Patients with Chiari malformation are often referred by a general practitioner with a diagnosis of chronic persistent neck pain, only to have the rehabilitation, specialist discover the malformation after obtaining a magnetic resonance imaging. Next, rehabilitation therapy is prescribed, including: medical, physical, and occupational therapy.

Medical therapy includes the use of analgesics, anti-inflammatory agents, and myorelaxants to reduce pain symptoms, which can have a mechanical rhythm or be of neuropathic type.

Physical therapy is also designed to relieve pain and to preserve the range of motion in the cervical spine joints and the shoulders.

The goal of occupational therapy is to give recommendations to the patient for the optimization of articular movements in order to reduce overloading of the upper limbs and neck, allowing the patients to continue their daily life activities (DLA) and work activities.

##### Physical therapy

The role of physical therapy in the multidisciplinary process of rehabilitation is the management of the patient's physical.

Taking into account the most frequent manifestations, the goals of physical therapy interventions include reduction of pain and spasticity, tone normalization, improvement of muscular activity and articular range of movement, balance and upright reactions re-education, and, facilitation of cervical spine, pectoral girdle, upper limbs, pelvic girdle, and lower limbs movements. There are different therapeutic options, physical therapy methods and techniques used to alleviate the pain: superficial and deep thermotherapy, electrotherapy, and massotherapy.

Methods that merit comment because alleviate motor disorders are: general kinesitherapy, postural treatment, specific kinesitherapy (proprioceptive neuromuscular facilitation [41], inhibition-facilitation of patterns of movement [42-44], cognitive therapeutic exercise [45]), balance and upright reactions re-education techniques (re-education of the cerebellar syndrome), gait re-education techniques, orofacial function, hydrotherapy, and balneotherapy.

The objective of physical therapy is to preserve and/or recover the patient's autonomy, reduce dependence, and improve quality of life. The patient should also consider certain behaviors and attitudes, some to follow and others to avoid.

##### Speech therapy

Craniocervical malformations can cause speech disorders as a result of paralysis, weakness, or incoordination of the speech musculature, with motor and functional disorders that affect phonation, resonance, articulation, and prosody [46].

Speech, swallowing, phonation, and respiration are functions that may be disturbed in people with Chiari syndrome and syringomyelia. They are part of the rehabilitation realm of the speech therapist. In children, in addition to the functional disorders mentioned above, language development and learning may be affected. Speech therapy has two types of intervention: preventive and palliative.

When a deviation from normal function is detected, even if it is mild, the patient and family members must be informed about the difficulties that may occur as the condition progresses. Reinforcement of swallowing, respiration, vocal tension and reading skills is important to preserve such functions. If no rehabilitation is initiated one problem can lead to another. For example, buccofacial motricity disorders can lead not only to swallowing disorders, but also to chewing and phonation abnormalities. Once the lesion is established, speech therapist interventions are palliative, targeting the areas where dysfunction is identified.

##### Craniosacral osteopathy techniques

Craniosacral osteopathy is another manual therapeutic resource in the management of Chiari syndrome; its purpose is to minimize symptoms and to improve the quality of life of the patient. In its minimal expression, the therapeutic approach has two basic principles; integrating vertebral biomechanics and normalizing tissular biodynamics, which as a whole, form an integrated functional unit whose components influence and interact with each other in the craniosacral system.

The anatomical and mechanical disturbances characteristic of Chiari syndrome in the craniocervical junction have a compressive mechanical effect on the dural membrane in its passage through the foramen magnum. They contribute to a modification in the hydrodynamic values of the Intracranial Pressure (ICP) and explain the broad array of symptoms associated to Chiari syndrome, which are related with the sensory organs, sensitivity, stability, balance, and motricity.

The goals for craniosacral osteopathy are to eliminate the restrictions that oppose or limit normal CSF flow, in order to maintain constant hydrodynamic values, allowing an efficient supply to tissues and nerves to conserve homeostatic integrity.

Interventions on the biomechanics of the craniosacral axis and actions on connective tissue restrictions and the extracellular matrix, where all the enzymatic metabolic and stimulus transmission processes take place [47], are the therapeutic bases of craniosacral osteopathy in Chiari syndrome.

## Psychological issues

The human being is conceived as a global being integrated by biological and psychological components in constant interaction with their environment. In this sense, chronic conditions, like Chiari syndrome and syringomyelia, involve a situation of change in which all the components of a person's health are implicated.

The World Health Organisation (WHO) defines health as “a state of complete physical, mental and social well-being, and not just the absence of disease or illness.” The introduction of the social factor as an element that configures the state of well-being, together with the physical and mental factors, means that psychosocial care is needed to cope with a health problem like Chiari syndrome and syringomyelia.

Therefore, a combined medical and psychological intervention is required for each patient, with the major objectives of reducing the psychosocial impact of the disease and improving the patient’s quality of life. In this context, psychotherapy is a therapeutic element that should be considered mandatory in the comprehensive management of patients with Chiari syndrome and syringomyelia. From this perspective, there are therapeutic options that reduce the psychosocial impact caused by the disease and improve the patient’s quality of life.

### Social and health ssues

The management of craniocervical malformations should begin with a correct and adequate diagnostic orientation and care of the patients, with a complete medical history and physical examination. Given the variability of their clinical expression, these malformations usually are not diagnosed in primary care centres, which are the natural gateway to the health-care system. They are identified primarily in specialized care or emergency rooms, where the patient is seen in episodes of disease exacerbation, since this conditon is usually oligosymptomatic or asymptomatic in early stages.

The patients, in addition to an adequate diagnosis, usually require individualized health-care plans, as well as a prolonged follow-up of the disease. This care should be based on a trained effective social and health-care team, that includes health-care professionals and social services, with the active participation of the patient's social network [48,49].

Globally, the responsibilities of the general practitioner (GP) regarding patients with syringomyelia and/or Chiari syndrome are similar to those related to any patient with low prevalent chronic diseases. Once diagnosed, the task is to “accompany” the patient, attend to their needs and associated problems, and assess any signs of worsening of the condition [50].

Thus, two phases can be identified regarding the relationship between the GP and the patient with syringomyelia and/or Chiari syndrome: the pre-diagnostic phase and the post-diagnostic phase.

The pre-diagnostic phase depends almost exclusively on imaging studies, in this case, MRI. This fact makes the condition practically undiagnosable in the primary care clinic. Suspected patients have to be referred to a neurology / neurosurgery clinic, where they can be assessed and the presence or absence of the condition established.

Once the diagnosis is made, patients should be reassured and, above all, informed that their symptoms are forms of expression of a possible craniocervical junction malformation, which in many cases can be corrected surgically. Also, control of the symptoms with the appropriate pharmacological therapy is critical. Based mostly on analgesia, in the first stages of the condition it is important to treat pain adequately.

Another professional closely linked to these patients after diagnosis is the physiotherapist. The social worker also is a key figure in the multidisciplinary approach to the care of these patients in health centres. For the general practitioner, referral of these patients to the social worker should not just be a way of avoiding problems within the patient's social environment, but a way to share the load of care with these professionals.

Finally, in dependent patients it is necessary to develop home care programmes, including the general practitioner, nurse, physiotherapist, and social worker. Although these patients comprise a small percentage of the total number of people with this syndrome, it is in these patients where primary care physicians must make an extra effort to address their needs [51].

### Role of the community

It is important to highlight the existence of advocacy groups that form a network with, among other objectives, the goal of disseminating health information to the community in order to increase the understanding, not only of this condition, but of many others, through conferences, seminars, or awareness campaigns at local and general levels. The dissemination of information related to craniocervical malformations from both health and social standpoints fulfill the need, often demanded by community members, for information on these diseases in particular, and on all Rare Diseases (RDs) in general. Information is considered as the first step in training and the keystone to any educational programme.

The educational programme referred above consists of providing information and training to the professionals involved in different aspects in the care of patients with craniocervical malformations, fundamentally health-care and social personnel (primary care doctors, specialists, nurses, physiotherapists, speech therapists, psychologists, social workers, etc.).

### Legal issues

Advising a person with craniocervical malformations regarding medical and legal issues can be very complex, and should be undertaken by a qualified and experienced specialist. The role of the medical professional is to provide up-to-date information, such as details of referrals and recommendations for other professionals, investigations developed, and recommended therapies [20].

As mentioned before, this can be a disabling condition, and may cause full disability in some patients, often requiring legal support to claim economic aid, special occupational conditions, occupational disability, and disability degree certification.

Work position adaptations, employment plans and promotion, insertion in the workplace or reinsertion after a prolonged sick leave, regardless of the worker's preparation and professional competence, are rights according to the legislation in force, but only if the degree of disability is 33% or more. Sometimes, this right is negotiated through collective agreements, as a result of disease or permanent total disability.

## Conclusions

It is important to determine the needs of the patients in terms of health-care, social, educational, occupational, and relationship issues, in addition to those derived from information aspects, particularly at onset of symptoms.

It is critical and necessary for the patient to receive adequate and validated information from the professionals who will be caring for them, so it is necessary that health-care professionals improve their communication skills and provide information to the affected people.

Since craniocervical malformations occur with diverse symptoms and an irregular course, these changing features are the factors which complicate most the timely diagnosis. Few primary care physicians are fully aware of these diseases and are capable to establish the diagnosis. It is necessary that professionals, especially primary care physicians, learn to identify the most characteristic symptoms of the most frequent craniocervical malformations (Chiari type I and syringomyelia) in order to develop a diagnostic suspicion and refer appropriately patients for diagnostic confirmation.

On the other hand, it is necessary that the physicians involved in the care of people with this condition comprehensively approach the management and follow-up of the patients, and that they organize interdisciplinary teams including all the professionals that can help to increase the quality of life of patients. In these teams, physiotherapists, rehabilitation medical specialists, pain specialists, psychologists, etc. have a special role.

The usefulness of different complementary support therapies should be verified, and should be included in the portfolio of services of the public health system whenever it is demonstrated that their use is beneficial for the patient.

Finally, another aspect to highlight is the existing legislation and regulations that should be adapted to consider these diseases as a possible cause of disability and discapacity.

## Competing interests

The authors declare that they have no competing interests.
